# 急性淋巴细胞白血病单倍体造血干细胞移植后中枢神经系统并发症1例

**DOI:** 10.3760/cma.j.issn.0253-2727.2023.04.017

**Published:** 2023-04

**Authors:** 晓亮 刘, 明 张, 业辉 谭, 欣 赵, 素君 高

**Affiliations:** 1 吉林大学第一医院血液科，长春 130021 Department of Hematology, the First Hospital of Jilin University, Changchun 130021, China; 2 吉林大学第一医院儿科，长春 130021 Department of Pediatrics, the First Hospital of Jilin University, Changchun 130021, China

患者，男，17岁，急性淋巴细胞白血病-普通B细胞型（标危组），以VDCLP方案诱导化疗达完全缓解，CAML、HD-MTX方案3疗程，VCLP方案1疗程，CAM方案1疗程，CAML方案1疗程巩固治疗，其后6-巯基嘌呤+甲氨蝶呤方案维持治疗，每半年给予VDLP方案化疗1疗程，于2015年11月维持治疗结束，共行腰穿及鞘内注射13次。规律复查持续完全缓解，微小残留病（MRD）阴性。2016年7月复查骨穿提示复发（幼稚淋巴细胞占15％），流式细胞术检测B系幼稚细胞占12.79％，染色体正常核型。于2016年8月行单药氯法拉滨治疗（52 mg/m^2^，第1～5天）达完全缓解，流式细胞术检测MRD 0.31％，继续单药氯法拉滨巩固1疗程，MRD阴性。2016年10月行单倍体造血干细胞移植（母亲供者），移植前行预防性鞘内注射1次，脑脊液检查未见异常。预处理方案为改良白消安/环磷酰胺+兔抗人胸腺细胞免疫球蛋白，应用环孢素A（CsA）、霉酚酸酯（MMF）及甲氨蝶呤预防移植物抗宿主病（GVHD），输注单个核细胞6.533×10^8^/kg，CD34^+^细胞4.217×10^6^/kg，移植后13 d血小板植入，17 d粒系植入，无急性GVHD表现，未发生严重感染。移植后100 d开始CsA减量（每周减量10％），规律随访骨髓完全缓解、MRD阴性、完全供者嵌合。

移植后180 d，CsA减量至10 mg/d，肝功能检查示天冬氨酸氨基转移酶（AST）280 U/L（参考值9～50 U/L）、丙氨酸氨基转移酶（ALT）617 U/L（参考值15～40 U/L）、γ-谷氨酰转移酶（γ-GT）646 U/L（参考值10～60 U/L）、碱性磷酸酶（ALP）317 U/L（参考值45～125 U/L）、总胆红素（TBIL）30.7 µmol/L（参考值0～26.0 µmol/L）、直接胆红素（DBIL）13.1 µmol/L（参考值0～6.8 µmol/L），乙型肝炎病毒DNA及丙型肝炎病毒RNA检测阴性，临床诊断：慢性GVHD中度（肝脏2分）。给予泼尼松1.0 mg/kg、CsA 70 mg/d口服。2周后再次复查肝功能检查示AST 104 U/L、ALT 151 U/L、γ-GT 534 U/L、ALP 267 U/L、TBIL 53 µmol/L、DBIL 32 µmol/L，腹部超声未见异常，复查CsA谷浓度为40 ng/ml，诊断为重度慢性GVHD（肝脏3分），将CsA增量至170 mg/d静脉滴注，血浓度维持200～250 ng/ml，继续CsA联合泼尼松治疗1个月，复查肝功能示AST 150 U/L、ALT 119 U/L、γ-GT 628 U/L、ALP 140 U/L、TBIL 167 µmol/L、DTIL 93 µmol/L，考虑慢性GVHD一线治疗效果不佳，加用MMF 30 mg/kg口服。

移植后239 d患者突发癫痫持续状态，予镇静后行头部MRI检查：双侧额顶枕叶、小脑半球见多发斑片状、脑回样异常信号（图1A），CsA 170 mg/d静脉滴注（血浓度为270 ng/ml）；脑脊液生化：蛋白0.80 g/L（参考值0.15～0.45 g/L），氯122 mmol/L（参考值119～129 mmol/L），葡糖糖3.3 mmol/L（参考值2.3～4.1 mmol/L），WBC 1×10^6^/L（参考值0～8×10^6^/L），脑脊液脱落细胞流式细胞术分析未见白血病细胞，脑脊液CMV-IgG抗体阳性，脑脊液CMV、EBV、HHV6均为阴性，脑脊液墨汁染色及培养阴性，乳酸脱氢酶正常，外周血涂片未见破碎红细胞，脑电图可见异常脑电波型，考虑可逆性后部脑病综合征（PRES），停用CsA，予以左乙拉西坦口服，同时给予甲泼尼龙250 mg每日2次静脉滴注（每3天甲泼尼龙剂量减半，直至停用）。期间无癫痫表现。移植后247 d复查肝功能示AST 524 U/L、ALT 343 U/L、γ-GT 1407 U/L、ALP 277 U/L、TBIL 218 µmol/L、DBIL 97 µmol/L，加用他克莫司静脉滴注（血浓度维持10 ng/ml），监测肝功转氨酶好转，胆红素进行性升高（TBIL 354 µmol/L，DBIL 214 µmol/L），加用5次巴利昔单抗并联合间充质干细胞输注5次（每周2次，每次1.0×10^6^/kg），TBIL降至36 µmol/L，未再发生癫痫表现。治疗过程中应用米卡芬净50 mg/d预防真菌感染。

移植后286 d出现左侧肢体活动不灵，下肢肌力减弱，无发热及头痛，无颈项强直，头部MRI增强扫描示枕叶、小脑半球白质病灶消失，右侧基底节区、放射冠及左侧额叶占位性病变，增强扫描见环形强化，大小为1.6～1.7 cm，周围见水肿带影（图1B）。脑脊液生化：蛋白0.91/L，氯121.3 mmol/L，葡糖糖3.4 mmol/L，WBC 1×10^6^/L，脑脊液脱落细胞流式细胞术MRD阴性，巨细胞病毒、EB病毒、人疱疹病毒6型DNA检测阴性，墨汁染色、细菌培养及半乳甘露聚糖试验均阴性，考虑颅内感染（真菌可能性大）。继续以他克莫司（血浓度10 ng/ml）治疗慢性GVHD，加用美罗培南、伏立康唑静脉滴注，治疗2周后复查头部MRI示“占位变化不明显”，停用美罗培南，伏立康唑改为口服，治疗4周后复查MRI头部病灶大小为1.0～1.4 cm，治疗100 d后左侧肢体活动正常，MRI示头部病灶消失。

移植后395 d患者自觉头痛、间断恶心、呕吐，头部CT示蛛网膜下腔出血（图1C）。血常规：WBC 4.96×10^9^/L，中性粒细胞计数4.02×10^9^/L，HGB 123 g/L，PLT 56×10^9^/L；凝血检查正常。头部CT血管成像（CTA）示右侧大脑后动脉、左侧前下动脉、右侧大脑中动脉起始部见囊性凸起影，脑血管造影显示颅内多发动脉瘤（图1D、E、F），终因脑出血加重而死亡。

讨论：该患者在移植后相继发生可逆性后部脑病综合征（PRES）、颅内侵袭性真菌病（IFD）、蛛网膜下腔出血（SAH）、感染性颅内动脉瘤（IIA），考虑与应用大剂量糖皮质激素及免疫抑制剂有关。

**图1:**
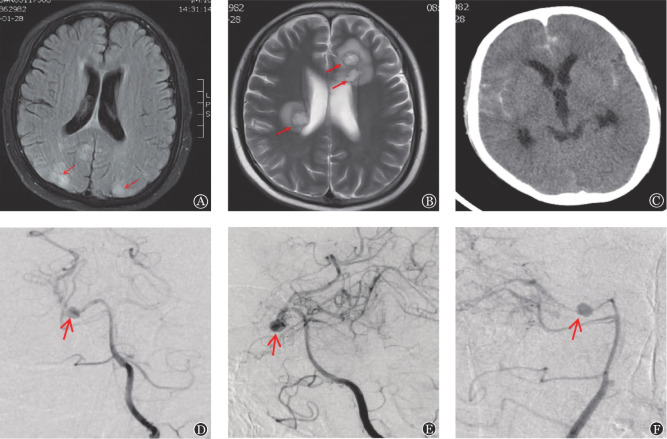
患者allo-HSCT后多次并发中枢神经系统病变影像检查 **注** A：移植后239 d可逆性后部脑病综合征MRI影像（双侧额顶枕叶、小脑半球见多发斑片状、脑回样异常信号）；B：移植后286 d颅内真菌感染MRI影像；C：移植后395 d蛛网膜下腔出血CT影像；D、E、F：移植后395 d脑血管造影示颅内多发动脉瘤形成

